# Comparison of Nutritional Quality of the Vegan, Vegetarian, Semi-Vegetarian, Pesco-Vegetarian and Omnivorous Diet

**DOI:** 10.3390/nu6031318

**Published:** 2014-03-24

**Authors:** Peter Clarys, Tom Deliens, Inge Huybrechts, Peter Deriemaeker, Barbara Vanaelst, Willem De Keyzer, Marcel Hebbelinck, Patrick Mullie

**Affiliations:** 1 Faculty of Physical Education and Physiotherapy, Department of Human Biometrics and Biomechanics, Vrije Universiteit Brussel, Pleinlaan 2, Brussels 1050, Belgium; E-Mails: tom.deliens@vub.ac.be (T.D.); peter.deriemaeker@vub.ac.be (P.D.); mhebbel@vub.ac.be (M.H.); patrick.mullie@vub.ac.be (P.M.); 2 Erasmus University College, Laerbeeklaan 121, Brussels 1090, Belgium; 3 International Agency for Research on Cancer (IARC), Dietary Exposure Assessment Group, 150 Cours Albert Thomas, Lyon 69372 CEDEX 08, France; E-Mail: Huybrechtsi@iarc.fr; 4 Faculty of Medicine and Health Sciences, Department of Public Health, Ghent University, De Pintelaan 185, Ghent 9000, Belgium; E-Mails: inge.huybrechts@ugent.be (I.H.); Barbara.Vanaelst@UGent.be (B.V.); willem.dekeyzer@ugent.be (W.D.K.); 5 Faculty of Science and Technology, Department of Bio- and food sciences, University College Ghent, Keramiekstraat 80, Ghent 9000, Belgium; 6 International Prevention Research Institute (iPRI), 15 chemin du Saquin, Ecully 69130, France

**Keywords:** vegan, vegetarian, omnivore, diet quality, dietary pattern analysis

## Abstract

The number of studies comparing nutritional quality of restrictive diets is limited. Data on vegan subjects are especially lacking. It was the aim of the present study to compare the quality and the contributing components of vegan, vegetarian, semi-vegetarian, pesco-vegetarian and omnivorous diets. Dietary intake was estimated using a cross-sectional online survey with a 52-items food frequency questionnaire (FFQ). Healthy Eating Index 2010 (HEI-2010) and the Mediterranean Diet Score (MDS) were calculated as indicators for diet quality. After analysis of the diet questionnaire and the FFQ, 1475 participants were classified as vegans (*n* = 104), vegetarians (*n* = 573), semi-vegetarians (*n* = 498), pesco-vegetarians (*n* = 145), and omnivores (*n* = 155). The most restricted diet, *i.e.*, the vegan diet, had the lowest total energy intake, better fat intake profile, lowest protein and highest dietary fiber intake in contrast to the omnivorous diet. Calcium intake was lowest for the vegans and below national dietary recommendations. The vegan diet received the highest index values and the omnivorous the lowest for HEI-2010 and MDS. Typical aspects of a vegan diet (high fruit and vegetable intake, low sodium intake, and low intake of saturated fat) contributed substantially to the total score, independent of the indexing system used. The score for the more prudent diets (vegetarians, semi-vegetarians and pesco-vegetarians) differed as a function of the used indexing system but they were mostly better in terms of nutrient quality than the omnivores.

## 1. Introduction

Vegetarian and semi-vegetarian diets are increasingly popular. Even the more restrictive vegan diet, with its exclusion of meat, fish, dairy and eggs, is gaining more and more popularity, especially amongst younger people [1]. Although several favorable health consequences are attributed to the vegan diet, concerns regarding the completeness of this restrictive dietary pattern still remain [2]. Indeed, there exists a perception that vegetarian, and especially the vegan diet, are deficient for important nutrients including protein, calcium, iron and vitamin B-12 [3,4,5,6,7,8].

A recent approach to assess the overall dietary quality is the use of indices analyzing a dietary pattern instead of the more reductive nutrient methodology [9]. As reviewed by Hu [9], dietary pattern analysis is a complimentary method to examine the effect of an overall diet: food and nutrients are not eaten in isolation, and the “single food or nutrient” approach will not take into account the complex interactions between food and nutrients. The Healthy Eating Index (HEI) represents the degree to which a dietary pattern conforms the official guidelines summarized in the United States Department of Agriculture Food Guide Pyramid, using a 10 or 12 component score [10,11,12]. The most recently released HEI (HEI-2010) uses an energy-adjusted approach, limiting the possible confounding effect of total energy intake [12]. The Mediterranean Diet Score (MDS) uses 10 components to express the agreement with the Mediterranean dietary pattern by 7 desirable and 2 undesirable components (meat and dairy) and 1 moderation (alcohol) component [13]. The necessity of specific components in the MDS (e.g., fish) makes this index less amenable for restrictive diets. In contrast, the HEI-2010 does not require a single commodity in order to obtain a high score [11,12].

The number of studies using dietary quality indices to compare restrictive diets with omnivorous diets is limited. The results of these studies are equivocal and none of them included a separate group of vegan subjects [7,10,14]. One study reports [15] the use of an Alternate Healthy Eating Index (AHEI) to examine the nutritional adequacy and quality of a low-fat vegan diet compared to a more conventional diabetes diet in type 2 diabetes patients. Patients switching to the low fat vegan diet significantly improved their AHEI score in every food category with a substantial increase for the fruit and vegetable components. Patients switching to the conventional diabetes diets did not improve their AHEI score [15]. The increase for the AHEI score was accompanied with a significant improvement of intake of several nutrients. Moreover, this study reported that the mean vitamin B-12 intake of the low fat vegan diet remained within the recommended range even without supplement use. This was likely due to the inclusion of several vitamin B-12 fortified foods in the diet.

The literature on index scores for a vegetarian diet remains equivocal, whilst to the best of our knowledge no indexing information is available for healthy subjects adhering to a vegan diet.

The aim of the present study was to analyze and compare the nutrient intake and the diet quality of vegans, vegetarians, semi-vegetarians, pesco-vegetarians and omnivorous subjects at least 20 years old and living in the Flemish part of Belgium. The HEI-2010 and MDS were calculated based on an online food frequency questionnaire (FFQ). Nutrient intake, total and component scores of the indices were analyzed as a function of the followed diet.

## 2. Experimental Section

### 2.1. Methods

Study Design and Population

A cross-sectional online food consumption survey was launched in 2012 and ran from February 2012 until April 2012. In order to reach a sufficient number of vegan and vegetarian participants, collaboration was established with “Ethisch Vegetarisch Alternatief” (Ethical Vegetarian Alternative) (EVA), an organization providing information about vegetarianism, known for the Flemish campaign of “Thursday Veggie Day” [16]. The EVA mailing list (including all the EVA members, *i.e.*, *n* = 4000) was one of the sampling frames. In order to reach also a sufficient number of omnivores, a mailing was also created through Ghent University and Ghent University College staff members (all grades). There were no specified exclusion criteria. All the invitees received an e-mail with an invitation letter to participate in the survey and could click on a link to enter the online questionnaire (see more details below).

This study was conducted according to the guidelines laid down in the Declaration of Helsinki. The Bioethical Committee of the Vrije Universiteit Brussel approved all procedures involving human subjects.

### 2.2. Assessments

#### 2.2.1. Food Frequency Questionnaire (FFQ)

A 52-item qualitative FFQ was developed based on the validated 50-items FFQ that was used in the Belgian Food Consumption Survey in 2004 [17]. However, extra items (products such as humus, tofu, quorn and fortified cereals, and fortified soy drinks) that are typically used by vegans and vegetarians were included to assure that also the protein sources of vegans/vegetarians were assessed. This FFQ included nine different frequency categories ranging from never to more than three times per day. The timeframe of this FFQ concerned the past year.

Operationalization of the FFQ was done by converting the consumption frequency values into frequency of daily intakes for all items. The midpoint of each consumption frequency category was used as the most probable consumption.

Energy and macronutrient intakes, as well as sodium, calcium and iron intakes were calculated by multiplying the daily frequency of specific foods by a standard portion (as proposed by the Superior Health Council) and by the amount of nutrient present in one gram [18].

The daily nutrient intake was calculated by summing up the nutrient content of each food item. The procedure was automatized using Statistical Package for Social Sciences 18.0 (SPSS, Inc., Chicago, IL, USA) scripts with data from a Belgian food composition table [19].

#### 2.2.2. Diet Questionnaire

Participants were also asked to identify their current diet: vegan (not consuming any animal products), vegetarian (not consuming any meat or fish), semi-vegetarian (consuming red meat, poultry or fish no more than once a week), pesco-vegetarian (consuming no meat but fish), and omnivorous (eating meat or fish almost every day). Subjects were classified in different diet groups using information from the diet questionnaire and the FFQ.

#### 2.2.3. Socio-Demographical and Weight/Height Information

Subjects were asked to enter their year of birth, gender and educational level online (no degree, lower education, secondary education, higher education, university degree or higher). In addition, subjects were also asked to enter their weight (kg) and height (cm).

### 2.3. Statistical Analysis

Of the 1803 participants, 328 (18%) were excluded because of missing values (*n* = 172) and age limitation (under 20 years of age) (*n* = 156). Subjects describing themselves as vegans in the diet questionnaire, though declaring to consume animal products in the FFQ as well as reported vegetarians declaring to consume meat as indicated by the FFQ were reclassified according to their answers given in the FFQ.

Descriptive statistics, such as frequencies and percentages were calculated for characterization of the participants (*i.e.*, gender; age stratified in 20 to 29 years, 30 to 39 years, 40 to 49 years, 50 to 59 years and 60 to 69 years; Body Mass Index (BMI) classified according to the World Health Organization in underweight BMI (*i.e.*, <18.5 kg/m^2^), normal weight (*i.e.*, 18.5 ≤ BMI < 25.0 kg/m^2^), overweight (*i.e.*, 25.0 ≤ BMI < 30.0 kg/m^2^) and obesity (*i.e.*, BMI ≥ 30.0 kg/m^2^), [20]; and educational level dichotomized in university or university college *versus* other.

The HEI-2010 and the MDS were computed as described previously [10,11,12,13]. The possible scores for the HEI indices ranged from 0 to 100 and for MDS from 0 to 9, with higher scores reflecting higher adherence to respectively the Food Guide Pyramid and the Mediterranean Diet. Means and standard deviations of the total and component scores for the HEI-2010 were calculated for vegans, vegetarians, semi-vegetarians, pesco-vegetarians and omnivores as well as a total score, numbers and percentage of the component scores for the MDS. For the HEI, total and component scores were compared using Analysis of Variance (ANOVA). Due to the nature of the MDS components (a 0 or 1 score based on the population specific medians), the ANOVA procedure could only be applied for the total score of the MDS. The abovementioned ANOVA procedures were followed by post hoc comparisons between the different diets (Bonferroni).

A two-sided 0.05 level of significance was defined, except for the post hoc tests where a significance level of 0.01 was set in order to reduce the possibility of a false positive result. SPSS 18.0 (SPSS Inc., Chicago, IL, USA) statistics software was used.

Normality of data was checked visually and with Kolmogorov-Smirnov test. Non-normal data distributions were tested with appropriate non-parametrical statistical tests. The results did not differ from parametrical tests, for uniformity of presentation and for clarity; only the results of parametrical tests were presented.

## 3. Results

In total, 1475 persons were included in the study, three out of four were females and almost 50% were less than 30 years of age (Table 1). Hundred and four persons were following a vegan diet (7.1%), 573 (38.8%) were vegetarians, 498 (33.8%) declared to be semi-vegetarians, 145 (9.8%) were pesco-vegetarians and 155 (10.5%) were omnivores. The percentages of participants with normal weights varied from 78.8% for vegans to 67.7% for omnivores; 8.7% of vegans were underweight, which was comparable with vegetarians and pesco-vegetarians. The prevalence of overweight and obesity was the highest for the omnivores, respectively 20.6% and 8.4%, and lowest for vegans (respectively 10.6% and 1.9%). Almost 80% of the sample had a university or university college level of education.

Table 2 presents the mean (SD) intake of macro- and micronutrients across the different diets. Vegans had a lower energy intake compared to other diets. In addition, the vegetarians had a significantly lower energy intake compared to the omnivores. No differences were detected when comparing the energy intake of respectively vegetarians, semi-vegetarians and pesco-vegetarians. Similar contrasts were found for total fat consumption, saturated and mono-unsaturated fat, dietary cholesterol, dietary proteins, alcohol, and sodium: lowest intakes were found in the vegan group compared with the omnivorous group (all *p* < 0.01), whilst differences between the more prudent diets were absent or less pronounced. The daily intake of saturated fat in vegans was 21 g (SD = 11) a day compared to 54 g (SD = 25) for omnivores (*p* < 0.01). The consumption of dietary poly-unsaturated fatty acids, dietary fiber and iron was related with the degree of restriction, with the highest consumption for vegans and the lowest for omnivores. Again, values for these three nutrients were comparable for the prudent diets. Absolute carbohydrate and sugar consumption (g/day) did not differ across the diet groups, whilst relative intakes (E%) showed a clear ranking with higher intakes as a function of the restrictiveness of the diet.

The highest calcium consumption was found in semi-vegetarians and pesco-vegetarians, the lowest in vegans, with respectively 1470, 1470 and 738 mg/day. Omnivores had lower calcium intakes (1199 mg/day) compared to the vegetarians and the semi-vegetarians (*p* < 0.001).

Table 3 presents the total and component scores for the HEI-2010. The vegan diet obtained the highest total score and the omnivorous diet the lowest total score for the HEI-2010. The more prudent diets (*i.e.*, vegetarian, semi-vegetarian, pesco-vegetarians) obtained comparable total scores.

**Table 1:** Characteristics of the study subjects.

Characteristics	Categorization	Responders	
		*n*	%
Total		1475	100.0
Gender	Male	369	25.0
	Female	1106	75.0
Age in years	20–29	697	47.3
	30–39	391	26.5
	40–49	218	14.8
	50–59	126	8.5
	60–69	43	2.9
Dietary patterns	Vegans	104	7.1
	Vegetarians	573	38.8
	Semi-vegetarians	498	33.8
	Pesco-vegetarians	145	9.8
	Omnivores	155	10.5
Underweight (<18.5 kg/m^2^)	Vegans	9	8.7
	Vegetarians	51	8.9
	Semi-vegetarians	31	6.2
	Pesco-vegetarians	12	8.3
	Omnivores	5	3.2
Normal weight (≥18.5 to <25.0 kg/m^2^)	Vegans	82	78.8
	Vegetarians	418	72.9
	Semi-vegetarians	370	74.3
	Pesco-vegetarians	105	72.4
	Omnivores	105	67.7
Overweight (≥25.0 to <30.0 kg/m^2^)	Vegans	11	10.6
	Vegetarians	84	14.7
	Semi-vegetarians	85	17.1
	Pesco-vegetarians	23	15.9
	Omnivores	32	20.6
Obesity (≥30.0 kg/m^2^)	Vegans	2	1.9
	Vegetarians	20	3.5
	Semi-vegetarians	12	2.4
	Pesco-vegetarians	5	3.4
	Omnivores	13	8.4
Educational level	University or university college level	1155	78.3

**Table 2:** Nutritional intake across the dietary patterns (*n* = 1475).

Macro- and Micronutrients	Vegans		Vegetarians		Semi-Vegetarians		Pesco-Vegetarians		Omnivores		*p*_anova_
	Mean	SD	Mean	SD	Mean	SD	Mean	SD	Mean	SD	
**Absolute Intake**											
Total energy (kcal)	2383 ^a,b,c,d^	804	2722 ^a,e^	875	2849 ^b^	858	2744 ^c^	797	2985 ^d,e^	1029	<0.001
Total fat (g)	68 ^a,b,c,d^	36	96 ^a,e^	43	107 ^b,e,f^	45	99 ^c,g^	39	122 ^d,f,g^	53	<0.001
Saturated fat (g)	21 ^a,b,c,d^	11	41 ^a,e,f^	21	47 ^b,e,g^	22	43 ^c,h^	19	54 ^d,f,g,h^	25	<0.001
Mono-unsaturated fat (g)	19 ^a,b,c,d^	12	31 ^a,e,f^	15	36 ^b,e^	17	32 ^c,g^	14	46 ^d,f,g^	21	<0.001
Poly-unsaturated fat (g)	28 ^a^	17	24	14	24	12	24	13	22 ^a^	11	<0.001
Cholesterol (mg)	149 ^a,b,c,d^	92	275 ^a,e,f^	125	321 ^b,e,g^	132	296 ^c,h^	111	376 ^d,f,g,h^	169	<0.001
Carbohydrates (g)	336	106	343	105	334	96	331	96	322	108	ns
Sugar (g)	156	61	162	65	155	60	154	52	149	60	ns
Fibers (g)	41 ^a,b,c,d^	14	34 ^a,b^	14	34 ^b,e^	12	33 ^c,f^	13	27 ^d,b,e,f^	10	<0.001
Proteins (g)	82 ^a,b,c^	39	93 ^d,e^	37	103 ^a,d^	36	100 ^b^	33	112 ^c,e^	45	<0.001
Alcohol (g)	7 ^a^	12	13 ^b^	17	11 ^c^	15	15 ^d^	19	21 ^a,b,c,d^	22	<0.001
Sodium (mg)	1316 ^a,b,c,d^	666	2228 ^a,e,f^	1013	2679 ^b,e,g^	1156	2371 ^c,h^	1047	3296 ^d,f,g,h^	1525	<0.001
Calcium (mg)	738 ^a,b,c,d^	456	1465 ^a,e^	819	1470 ^b,f^	712	1470 ^c^	765	1199 ^d,e,f^	682	<0.001
Iron (mg)	23 ^a,b,c,d^	10	20 ^a,e^	8	20 ^b^	6	20 ^c^	7	17 ^d,e^	6	<0.001
**Nutrient Density**											
Total fat (E%)	25 ^a,b,c,d^	8	31 ^a,e,f^	7	33 ^b,e,g^	6	32 ^c,h^	7	36 ^d,f,g,h^	7	<0.001
Saturated fat (E%)	8 ^a,b,c,d^	3	13 ^a,e,f^	4	14 ^b,e,g^	4	14 ^c,h^	4	16 ^d,f,g,h^	3	<0.001
Carbohydrates (E%)	57 ^a,b,c,d^	8	51 ^a,e,f,g^	8	48 ^b,e,h^	7	49 ^c,f,i^	7	44 ^d,g,h,i^	8	<0.001
Sugar (E%)	27 ^a,b,c,d^	9	24 ^a,e,f^	7	22 ^b,e,g^	6	23 ^c^	6	21 ^d,f,g^	8	<0.001
Proteins (E%)	14 ^a^	4	14 ^b,c,d^	3	15 ^b^	3	15 ^c^	3	15 ^a,d^	3	<0.001
Sodium (mg/1000 kcal)	546 ^a,b,c,d^	202	815 ^a,e,f^	261	934 ^b,e,g^	272	859 ^c,h^	244	1095 ^d,f,g,h^	308	<0.001
Calcium (mg/1000 kcal)	306 ^a,b,c,d^	136	530 ^a,e^	212	512 ^b,f^	173	531 ^c^	204	406 ^d,e,f^	185	<0.001
Iron (mg/1000 kcal)	10 ^a,b,c,d^	2	8 ^a,e,f^	2	7 ^b,e,g^	2	7 ^c,h^	1	6 ^d,f,g,h^	1	<0.001

^a–h^ Dietary patterns with the same superscripts differ significantly in the post hoc test (*p* < 0.01).

**Table 3:** Total and component scores for Healthy Eating Index 2010 (*n* = 1475).

Healthy Eating Index 2010 Components	Vegans		Vegetarians		Semi-Vegetarians		Pesco-Vegetarians		Omnivores		*p*_anova_
	Mean	SD	Mean	SD	Mean	SD	Mean	SD	Mean	SD	
Total Fruit ^£^	4.5 ^a,b^	1.1	4.1 ^c^	1.4	3.9 ^a,d^	1.4	4.1 ^e^	1.3	3.2 ^b,c,d,e^	1.6	<0.001
Whole Fruit ^μ^	4.5 ^a^	1.2	4.1 ^b^	1.5	4.1 ^c^	1.5	4.1 ^d^	1.5	3.5 ^a,b,c,d^	1.8	<0.001
Total Vegetables	4.8	0.7	4.8	0.8	4.8	0.8	4.8	0.6	4.6	1.1	<0.05
Greens and beans	4.8 ^a,b^	0.7	4.6 ^c^	1.0	4.4 ^a,d^	1.1	4.5 ^e^	1.0	3.6 ^b,c,d,e^	1.7	<0.001
Whole Grains	7.0	3.4	7.6 ^a^	3.0	7.9 ^b^	2.8	7.3	3.0	6.8 ^a,b^	3.3	0.001
Dairy ^§^	0.0 ^a,b,c,d^	0.0	2.8 ^a^	2.2	3.2 ^b^	2.2	3.0 ^c^	2.1	3.0 ^d^	2.3	<0.001
Total proteins foods	4.0 ^a,b,c,d^	1.2	3.2 ^a,e,f^	1.3	3.4 ^b,e,g,h^	1.3	2.9 ^c,g,h,i^	1.3	4.4 ^d,f,i^	1.1	<0.001
Seafood and plant proteins	4.6 ^a,b,c^	1.1	3.5 ^a,d,e,f^	1.6	3.8 ^b,d,g,h^	1.3	4.3 ^e,g,i^	1.2	2.9 ^c,f,g,i^	1.5	<0.001
Fatty acids	7.5 ^a,b,c,d^	2.3	2.2 ^a,e,f^	2.8	1.6 ^b,e^	2.3	1.8 ^c^	2.4	1.6 ^d,f^	1.6	<0.001
Refined grains	5.5 ^a,b,c,d^	3.6	7.1 ^a,e^	3.0	4.5 ^b,f^	3.1	7.2 ^c,g^	3.3	8.5 ^d,e,f,g^	2.5	<0.001
Sodium	9.8 ^a,d,c,d^	0.5	8.7 ^a,e,f^	1.6	8.0 ^b,e,g^	1.8	8.5 ^c,h^	1.5	6.8 ^d,f,g,h^	2.1	<0.001
Empty calories *	8.5 ^a,b,c,d^	5.1	6.0 ^a,e^	3.9	6.9 ^b,e^	4.2	6.4 ^c,f^	4.1	5.7 ^d,f^	4.3	<0.001
**Healthy Eating Index 2010**	65.4 ^a,b,c,d^	8.3	58.7 ^a,e^	8.9	59.4 ^b,f^	7.4	58.7 ^c,g^	7.9	54.2 ^d,e,f,g^	9.0	<0.001

^a–i^ Diet groups with the same indices differ significantly in the post hoc test (*p* < 0.01). ^£^ Includes fruit juice. ^μ^: Includes all forms except juice. ^§^ Due to the structure of the FFQ dairy alternatives (e.g., soy beverages) were classified under “Total protein foods” and not as “Dairy”. * Calories from solid fats, alcohol, and added sugars; threshold for counting alcohol is13 g/1000 kcal.

Typical components of the vegan and vegetarian diets (*i.e.*, fruit, vegetables, low fat content, low sodium content) contributed to the high total score for these components, whilst the omnivorous diet resulted in the lowest scores for these components.

Vegans obtained a zero score for the milk and dairy component since the alternatives for these products were classified under the component protein sources.

The HEI-2010 delivered no difference for the seafood and plant proteins between the vegans and the pesco-vegetarians, whilst the omnivorous group received the lowest score for that component. The empty calories component resulted in the highest scores for the vegans and the lowest for the omnivorous group.

Table 4 presents the data for the MDS. The highest mean (SD) score was found for the vegans followed by the pesco-vegetarians. Both differed significantly from the omnivorous diet, which obtained the lowest total score (all *p* < 0.01). Concerning the prudent diets, the total score for the vegetarians was significantly lower compared to the semi-vegetarian and pesco-vegetarian diet. The percentage of subjects above the median intake was lower for the omnivores compared to those of the other diets for the vegetables, legumes, fruit, nuts, and cereals component.

**Table 4:** Distribution across components and total score for the Mediterranean Diet Score (*n* = 1475).

Mediterranean Diet Score Components	Vegans		Vegetarians		Semi-Vegetarians		Pesco-Vegetarians		Omnivores	
	*n*	%	*n*	%	*n*	%	*n*	%	n	%
Higher intake than median										
Vegetables	60	58	289	50	240	48	74	51	56	36
Legumes	87	84	424	74	339	68	98	68	66	43
Fruit	70	67	299	52	242	49	75	52	54	35
Cereals	71	68	305	53	254	51	72	50	43	28
Fish	0	0	0	0	460	92	143	99	142	92
Meat and meat replacement	48	46	373	65	235	47	112	77	24	16
Dairy	0	0	290	51	210	42	67	46	75	48
Nuts	75	72	299	52	207	42	63	43	23	15
Mono-unsaturated Fat/saturated fat	63	61	239	42	249	50	57	39	115	74
Ethanol	22	21	113	29	146	29	41	28	33	21
**Mediterranean Diet Score**	Mean	SD	Mean	SD	Mean	SD	Mean	SD	Mean	SD
	5.8 ^a,b,c^	1.3	4.6 ^a,d,e,f^	1.5	5.2 ^b,d,g^	1.5	5.5 ^e,h^	1.4	4.1 ^c,f,g,h^	1.6

^a–f^ Diet groups with the same indices differ significantly in the post hoc test (*p* < 0.01).

## 4. Discussion

In this study, the dietary indices HEI-2010 and MDS were calculated for subjects with different dietary patterns, ranging from the omnivorous to the more restrictive vegan diet. The purposeful sampling mainly obtained via the website of EVA allowed us to reach a considerable number of vegans who are often underrepresented in comparative studies. We could equally include different groups of totally or partially meat avoiders or so called prudent diets [8]: vegetarians, pesco-vegetarians and semi-vegetarians. Purposeful sampling was necessary since the prevalence of veganism and vegetarianism in Belgium is very low (*i.e.*, less than 1%) [21].

Except for the omnivores, all diet groups had a comparable number of underweight subjects (ranging from 6.2% to 8.9%), whilst this was only 3.2% for the omnivores. These percentages were reversed for overweight and obesity, with a higher prevalence of overweight and obese subjects amongst the omnivores compared to the other diet groups. These findings are in agreement with published literature, where pesco-vegetarians, vegetarians and especially vegans had lower BMI than meat-eaters [22].

Nutritional intake of vegans compared to an omnivorous diet is in line with earlier research on vegans. Indeed, the most restricted diet had lowest total energy intake, better fat intake profile (*i.e.*, lower cholesterol, total and saturated fat and higher poly-unsaturated fat), lowest protein and highest dietary fiber intake in contrast to the omnivorous diet. The intakes of the prudent diets were in between the vegan and omnivorous values. Absolute carbohydrate and sugar intakes were of the same magnitude across all diets, whilst relative intakes were highest in the vegan and lowest in the omnivorous diet. The higher carbohydrate intake as a function of the restriction results in a better macronutrient distribution for the more restrictive diets, which is in line with the literature [23]. It is well known that fruit is an important contributor of carbohydrates and sugars, especially in the more restricted diets, where fruit consumption is generally high [2]. Moreover, other common and less healthy sources of sugar (*i.e.*, candy, chocolate, cake and cookies) often contain animal products allowing only limited availability of these sugar sources for vegans [1]. Sodium intake in vegans is less than half of the omnivorous intake. Although not of the same magnitude, lower sodium intakes have been reported when comparing respectively vegetarian [7] and vegan diets [15] with omnivorous diets. The restrictive diets allowing dairy consumption had the highest calcium intakes with the vegans only reaching half of these values. Indeed, in Western countries, dairy products are a major source of calcium in most diets [24]. The study of Appleby and colleagues [4] points to the increased fracture risk in vegans compared to omnivorous, pesco-vegetarians and vegetarians. However, vegans with intakes above the United Kingdom estimated average reference intake of 525 mg/day did not show increased fracture risk. Mean vegans’ calcium intake in the present study (738 SD = 456 mg/day) was slightly above the reported values of the EPIC-Oxford vegans (603 SD = 232 mg/day for men; 586 SD = 226 mg/day for women). In agreement with the EPIC-Oxford study, a certain similarity was detected for the calcium intakes for omnivores, vegetarians, semi-vegetarians and pesco-vegetarians [4]. The iron intake, with the most favorable values for the vegans, will not automatically result in an optimal iron status, since absorption of non-haem iron is less efficient [2,8]. Analysis of the different components of the HEI-2010 and the MDS indicate that vegans obtained the better scores for vegetables and legumes. The study of Ball & Bartlett demonstrated the importance of the vegetables component when comparing the iron intake of vegetarian *versus* omnivorous women [25]. Our results are in line with those of the comparative study of Larsson & Johansson on vegan adolescents *versus* omnivores where vegan iron intake in females was significantly higher compared to their omnivorous counterparts [1]. The uneven gender distribution in our vegan sample (70% females) may partly explain these high iron intakes since dietary practices in women are generally better than those in men [25].

The highest HEI-2010 total scores were found for the vegans and the lowest for omnivores. The prudent diets (vegetarians, semi-vegetarians and pesco-vegetarians) obtained a score in between the restricted and the unrestricted diet groups. Discrimination between the different prudent diets was not possible using the HEI-2010. Using the MDS resulted in another ranking: vegans received the highest total score followed by the pesco-vegetarians, the semi-vegetarians, the vegetarians and the omnivores. In the study of Kennedy *et al.* [10], and using the HEI-1995, vegetarians received a lower total score compared to the omnivores whilst in the study of Farmer *et al.* [7] the non-dieting vegetarians obtained a higher score on the HEI-2005 compared to the omnivores. The study of Clarys *et al.* [14] reported equally higher scores for properly matched vegetarians compared to omnivorous subjects when using the most recently released version of the HEI and the MDS.

The discrepancy between the rankings obtained with the MDS compared to the HEI-2010 rank order may be caused by several factors. Firstly, scores in the MDS model are attributed based on the median value of different components. Hence, when health conscious populations are studied—as is the case in our self-selected sample—this method may lose discriminative power [26,27]. In addition, although both indices are hypothesis driven models, they attribute different health effects to specific components. Indeed, in the HEI milk is an “adequacy” component whilst it is an “undesirable” component in the MDS. Moreover, in the MDS specific commodities influence the diet groups in a different way. The absence of the MDS-desired component fish in the vegan and vegetarian diets negatively influences the score of these groups. This is partly counterbalanced by a positive score for the undesirable milk component for the vegans. The inclusion of fish with adequate meat replacement may explain the high score for the pesco-vegetarians in the MDS system whilst the moderate meat consumption for the semi-vegetarians results equally in a positive classification. The moderation components in the HEI-2010 (*i.e.*, refined grains, sodium, and empty calories) are not commodity driven and may be an indication for poorer choices within different components.

The used indexing systems allowed to discriminate between the restricted and the unrestricted diet groups. The most restrictive diet received the highest score whilst the omnivorous pattern received the lowest scores.

High scores in both indexing systems (HEI-2010 and MDS) are related with positive health outcomes [28,29] in a general population. Their different definition of an optimal diet and their different scoring mechanism resulted in different rankings of the prudent diets. Especially the possibility to include both animal and plant protein sources in the newer HEI-2010 makes it amenable to estimate the quality of restricted plant based diets. The use of traditional components such as meat and fish is a major drawback of the MDS system when working with meat and fish restricted or vegan diets. The latter explains the high score for the pesco-vegetarians when using the MDS.

However, it should be kept in mind that the indexing systems may be an indication for several healthy components of the diet while some specific nutrients may be suboptimal. Indeed, the used indices are for a large part based on macronutrient composition and sodium intake whilst some specific nutrients are not analyzed. The indices scores for vegans may be unrelated to vitamin B12 and vitamin D intake since it does not take into account the use of fortified products. The study of Larsson and Johansson found indeed lower intakes of both vitamins for the vegan subjects compared to the omnivorous subjects [1]. However, the study of Turner-Mc Grievy *et al.* [15], suggests sufficient consumption of fortified products in their vegan diet sample whilst Larsson and Johansson [1] advise only vitamin D supplement use in case of insufficient sun exposure. In order to take these important aspects of restrictive diets into account, indices should include specific questioning concerning fortified food items and/or supplement use.

An important strength of this study is its large sample of vegan and vegetarian diets, a population group that was importantly underrepresented in the Belgian Food Consumption Survey because of its low prevalence [21]. Another strength of this study is the use of a FFQ that has been developed, tested and validated for assessing food intake in the frame of the Belgian Food Consumption Survey in 2004 [17,21].

A limitation of this study is the fact that the study sample is not representative for the general population, as a convenient sample was used. From Table 1 it may be concluded that mainly the higher educated levels are overrepresented in comparison with representative surveys such as the Belgian Food Consumption Survey. However, it would not be possible to obtain sufficient power for all diet groups (more in particular for the vegan group) when using random sampling as restricted diets have been shown to be very low in our Belgian population [21]. Another limitation of this study is the use of self-reported weight and height information to calculate BMI and divide subjects in BMI-categories, which may underestimate the true BMI [30]. The limited items in the FFQ and the long recall period (1 year) have some disadvantages and this may weaken the discriminative power of the used FFQ. Indeed, the validation study of De Keyzer *et al.*, demonstrated poor ranking agreement for some food groups (e.g., bread and cereals, potatoes and grains) [17]. Uncertainty within these food groups, which are important components of plant based diet, may have influenced the score within both indexing systems. In addition, the structure of the FFQ, collecting information on food group level and not on food item level, did not allow to separate the different protein sources. As a consequence, all soy products were classified under the plant protein group resulting in a zero score for milk and dairy for the vegans and possibly a lower score for this component for the vegetarians consuming these products. The latter results in a zero score component score for milk and dairy among the vegans. The effect of this deviation from the HEI-2010 guidelines may be moderated in the total score since these products will positively influence the plant protein component. Portion sizes were not assessed but according to Molag *et al.* [31] the latter does not consistently influence the ranking of subjects using FFQs. Finally it is well known that FFQs tend to underestimate the level of energy and protein intake allowing no accurate intake assessment. Nevertheless, FFQs allow to distinguish between different subpopulations [31]. The results of the present study are in line with an earlier study on vegetarians where HEI-2010 and MDS were calculated for food intake as assessed by three day food diaries [14].

Our results together with others [14,15] indicate the robustness of the used indices to detect the diets with the highest number of healthy items. Future prospective research should study if the relations between these indices and health outcomes hold for restricted diets. Furthermore, sufficient attention should be given to possible confounders of the relation index score and health outcome since people on restrictive diets are often more health conscious compared to the general population. Finally, blood analysis in combination with specific questioning for supplements/fortified products use in vegans may indicate whether a high index score covers all aspects of a complete diet.

## 5. Conclusions

In conclusion, results concerning body weight, nutritional intake, nutritional quality and quantity are in line with the literature on restricted and prudent diets *versus* unrestricted omnivorous diets. The use of indexing systems, estimating the overall diet quality based on different aspects of healthful dietary models (be it the US Dietary Guidelines for Americans or the compliance to the Mediterranean Diet) indicated consistently the vegan diet as the most healthy one. The impossibility to score for (a) specific component(s) for the restricted diets was compensated by the higher scores on most of the other components. Adaptation with specific components (e.g., soy drinks instead of milk; inclusion of other polyunsaturated fat sources instead of fish) may increase the relation with different types of healthful diets, and this especially for the MDS system.

Nevertheless, the used indices may be useful as a screening method allowing the judgment of specific diets.
